# Nursing Home Compare Star Rankings and the Variation in Potentially Preventable Emergency Department Visits and Hospital Admissions

**DOI:** 10.1089/pop.2018.0065

**Published:** 2019-03-27

**Authors:** Richard L. Fuller, Norbert I. Goldfield, John S. Hughes, Elizabeth C. McCullough

**Affiliations:** ^1^ 3M Health Information Systems, Clinical and Economic Research, Silver Spring, Maryland.; ^2^ Brightwood Riverview Health Center, Springfield, Massachusetts.; ^3^ Yale University School of Medicine, New Haven, Connecticut.

**Keywords:** preventable admissions, nursing home care, emergency department visits

## Abstract

Measurement of the quality of US health care increasingly emphasizes clinical outcomes over clinical processes. Nursing Home Compare Star Ratings are provided by Medicare to help select better nursing home care. The authors determined the rates and types of 2 important clinical outcomes–potentially preventable hospital admissions and potentially preventable emergency department (ED) visits–for a subset of 439,011 long-term nursing homes residents residing in 12,883 nursing homes throughout the United States over a 2-year period (2010–2011) and compared them with the Star Rating system. This study found that (1) the likelihood of potentially preventable events increases with increasing burden of chronic illness, (2) the principle reasons for hospital admissions and ED visits (eg, septicemia, pneumonia, confusion, gastroenteritis) are not part of existing nursing home quality measures, (3) the rate of potentially preventable admissions and ED visits for nursing homes residents varies greatly both across and within states, with 5 states having in excess of 20% more than the national average for both, and (4) the Nursing Home Compare Stars measure has limited correlation with rates of these potentially preventable events. Nursing Home Compare Star rankings could benefit by incorporating outcomes measures such as preventable hospitalizations and ED visits, and by comparing nursing home performance on results drawn from across states rather than within them. Such reform could better help users find nursing homes of higher quality and stimulate homes to improve quality in ways that benefit residents.

## Background

There is considerable national interest in measuring the quality of health care outcomes. This is of particular importance for long-term stay residents of nursing homes who are both vulnerable and dependent on the care provided by their residential facilities. In the United States, 85% of long-term residents of nursing homes are older than 65 years of age and typically qualify for Medicare while the majority of those younger than age 65 typically qualify for Medicare through disability.^1^ Medicare benefits provide coverage for acute medical conditions that affect eligible beneficiaries while other payment sources (primarily Medicaid) are used to pay for the residential care itself. Thus, acute care events, such as hospitalizations and emergency department (ED) visits, paid via a separate payer source (Medicare) are monitored infrequently for nursing home quality because of the logistical challenges of matching claims data to residents. The study team identifies long-term nursing home residents from within a Medicare fee-for-service (FFS) claims database. The team then calculates 2 important clinical outcomes– risk-adjusted rates of potentially preventable hospital admissions (PPAs) and ED visits (PPVs)–for the residents' nursing homes from a nationwide sample. The study team next evaluates nursing home performance on PPAs and PPVs and their Star rankings assigned by the Centers for Medicare & Medicaid Services (CMS) to assess comparability. CMS created the 5-Star Rating System in 2008 as a guide to nursing home performance for patients and families. The overall Star ranking calculated by the system is dependent on the interplay of 3 domains: (1) reports from health inspectors who make site visits (survey metric); (2) case mix adjusted registered nurse and total nursing staffing hours per resident (staffing metric); and (3) quality scores derived from 16 of the 24 measures posted on the Nursing Home Compare website^2^ (quality metric).

Previous studies have estimated the volume of ED visits, the acuity of nursing home residents relative to other patients using the ED, and the propensity to be admitted via the ED.^3,4^ The nursing home INTERACT (Interventions to Reduce Acute Care Transfers) program has been piloted to reduce preventable hospitalizations and ED visits without specifying the nature and frequency of those encounters.^5^ The study team is unaware of any other national comparison of the frequency and cause of hospital encounters from nursing homes.

The present analysis risk adjusts for variation in the relative complexity of residents in one nursing home compared to another before comparing rates of PPAs and PPVs (without admission), which are specified so as to enable comparison of nursing home care management. The study team compares the frequency of these outcomes within and across states in order to examine their variability.

## Methods

This analysis was retrospective and not subjected to Institutional Review. Personal health information was managed so as to maintain the privacy of individuals in accordance with Health Insurance Portability and Accountability Act guidelines at all times. The study team obtained Medicare claims data for 28,303,570 FFS beneficiaries for the calendar years 2010 and 2011, including associated Minimum Data Set (MDS)^6^ assessment submissions. Nursing home residents enrolled in Medicare FFS were identified within the data extract. Those residents reported as dying in 2011 were excluded from analysis and, to help mitigate reporting errors (eg, failure to record death or switch to managed care), beneficiaries with no reported FFS claims in 2011 were excluded. MDS submissions were reviewed for these remaining beneficiaries between the period January 1, 2010, and January 31, 2011 to find matching facility submissions. The CMS certification number for the facility providing the last MDS submission matched to the beneficiary was assigned as the beneficiary's nursing home. Where a matching MDS submission was not found, the beneficiary was excluded from analysis, resulting in a final sample of 439,011 beneficiaries distributed across 12,883 nursing homes, drawn from a larger group of nursing home residents: in 2010 there were a reported 1,392,000 residents and 15,646 certified nursing homes^6^ in the United States. This includes residents who may have died in 2011 and those not enrolled with Medicare FFS.

The Medicare FFS claims data were analyzed using 3M Population-Focused Preventable software^7^ to determine rates of PPAs and PPVs for the beneficiaries. Nursing home PPAs are hospital admissions consisting of 62 out of 314 base All-Patient Refined Diagnosis Related Groups (APR DRGs)^8^ that were judged by a physician panel to possibly result from inadequate supervision or care (eg, medication management). Therefore, high rates of PPAs may represent a failure of nursing home care. Nursing home PPVs are ED visits (without subsequent admission) formed from a subset of Enhanced Ambulatory Patient Groups (EAPGs),^9^ a comprehensive catalog of outpatient encounters. Rates of PPAs and PPVs are risk adjusted using Aggregated Clinical Risk Groups (ACRGs),^10^ a classification system of mutually exclusive categories that stratifies individuals according to their profile of chronic health conditions and their expected use of health care resources. ACRGs make explicit recognition of the interaction of 2 or more chronic health conditions and the gradations of severity of illness within the underlying conditions, with the option to adjust for functional health status.^11^ The PPA and PPV rate methodology is used by several Medicaid programs to assess and compare the rate of hospital admissions and ED visits from the community.^12,13^

Each beneficiary was assigned to a single ACRG using claims data for 2010. PPAs and PPVs identified in 2011 were weighted in proportion to their standardized (relative) cost using weights calculated for APR DRGs (PPA) and EAPGs (PPV). The average 2011 weight associated with PPAs or PPVs for each ACRG is assigned as the expected weight for 2011 for any beneficiary in that ACRG classification. The actual PPA or PPV weight is the sum of the PPA or PPV weights for a beneficiary in 2011. In this way each beneficiary is assigned an expected weight (based on their 2010 ACRG assignment) and an actual weight based on the mix of PPAs and PPVs he or she experienced in 2011.

Nursing home Star Ratings were downloaded for the 2011 claims period from the CMS website.^14^ Each nursing home's monthly Star Rating was converted into an annual average and compared to the performance ranking obtained using patient-focused preventable events to assess similarity.

## Results

Table 1 reports the number of PPAs and PPVs for 2011.

**Table 1.** Rates of Potentially Preventable Hospital Admissions and Emergency Department Visits by Nursing Home Resident Aggregated Clinical Risk Group

*Aggregated clinical risk group description*	*Severity level*	NH residents at risk^*^	*PPA events per resident*	*PPV events per resident*
Healthy or history of significant acute disease		1485	0.065	0.180
Single Minor Chronic Disease	Level 1–2	974	0.045	0.139
Minor Chronic Disease in Multiple Organ Systems	Level 1–4	1327	0.041	0.091
Single Dominant or Moderate Chronic Disease	Level 1–2	11,127	0.047	0.106
Single Dominant or Moderate Chronic Disease	Level 3–4	2115	0.081	0.130
Single Dominant or Moderate Chronic Disease	Level 5–6	1586	0.082	0.180
Significant Chronic Disease in Multiple Organ Systems	Level 1–2	57,822	0.057	0.114
Significant Chronic Disease in Multiple Organ Systems	Level 3–4	117,250	0.088	0.151
Significant Chronic Disease in Multiple Organ Systems	Level 5–6	61,980	0.146	0.247
Dominant Chronic Disease in 3 or More Organ Systems	Level 1–2	21,705	0.091	0.154
Dominant Chronic Disease in 3 or More Organ Systems	Level 3–4	91,267	0.170	0.225
Dominant Chronic Disease in 3 or More Organ Systems	Level 5–6	41,559	0.298	0.380
Malignancy, Under Active Treatment	Level 1–3	2692	0.097	0.163
Malignancy, Under Active Treatment	Level 4–5	7227	0.225	0.314
Catastrophic Conditions	Level 1–3	5171	0.140	0.331
Catastrophic Conditions	Level 4–6	13,724	0.345	0.660
**ALL**		**439,011**	0.140	0.220

Source: Medicare Fee for Service enrollment FY 2010/2011. Potentially preventable event rates computed for FY 2011.

^*^ Nursing home residents at risk is the count of residents matched to a nursing home that may have none, one or more PPA or PPV events.

FY, fiscal year; NH, nursing home; PPA, potentially preventable hospital admissions; PPV, potentially preventable emergency department visits.

One can observe an average of 0.140 PPAs and 0.220 PPVs per nursing home resident. These rates are observed to increase with the complexity of the patient (ie, the rate is higher for higher levels of ACRG complexity and severity) with the separation between the ACRG group rate and the overall average most pronounced for those enrollees with 3 or more chronic diseases or catastrophic conditions, such as individuals on total parenteral nutrition. However, there are 3786 (0.86%) residents in the lowest levels of ACRGs who ordinarily would be considered “healthy” because of the absence of chronic conditions. This is unexpected for long-term nursing home residents, whose records probably were coded inadequately, and indicates that the use of matched claims data is efficient, but imperfect, in assigning clinical risk.

Table 2 presents the most frequently occurring types of PPAs and PPVs. Approximately 52% of hospital admissions and 51% of ED visits from nursing homes were identified as potentially preventable (potentially amenable to care management initiatives to reduce their rate). More than 80% of PPA event types are concentrated within 10 categories. In all, 57% of PPAs fall into 4 base APR DRGs: septicemia; major respiratory infections & inflammations; other pneumonia; and kidney and urinary tract infections (Table 2). The prevalence of nursing home hospitalizations related to septicemia has been reported previously by the Office of the Inspector General (OIG),^15^ with an estimated financial impact of approximately $3 billion.

**Table 2.** Most Common Nursing Home Potentially Preventable Hospital Admission and Potentially Preventable Emergency Departments Visit Event Types

*PPA*		
*APR DRG*	*Description*	*Pct of NH PPA*
720	Septicemia & disseminated infections	28.10%
137	Major respiratory infections & inflammations	9.70%
139	Other pneumonia	9.50%
463	Kidney & urinary tract infections	8.90%
194	Heart failure	5.70%
710	Infectious & parasitic diseases including HIV with operating room procedure	4.70%
140	Chronic obstructive pulmonary disease	4.60%
308	Hip & femur procedures for trauma except joint replacement	4.50%
133	Pulmonary edema & respiratory failure	3.90%
383	Cellulitis & other bacterial skin infections	2.80%
**Total**		82.50%

*PPV*		
*Category: Diseases and disorders of the digestive system [22.7%]*		
*EAPG*	*Description*	*Pct of PPV*
624	Level I Gastrointestinal diagnoses(eg, GI bleeding/hemorrhoids or complications of eating, digesting and swallowing)	8.23%
628	Abdominal pain	4.48%
629	Malfunction, reaction & complication of GI device or procedure	4.16%
627	Non-bacterial gastroenteritis, nausea & vomiting	3.87%
	Other	2.03%

*Category: Diseases and disorders of the kidney and urinary tract [18.08%]*		
*EAPG*	*Description*	*Pct of PPV*
727	Acute lower urinary tract infections	11.03%
726	Other kidney & urinary tract diagnoses, signs & symptoms (eg, complications of urinary management, discomfort)	3.64%
	Other	3.40%

*Category: Rehabilitation, aftercare, other factors influencing health status and health services [11.8%]*		
*EAPG*	*Description*	*Pct of PPV*
871	Signs, symptoms & other factors influencing health status (eg, weakness, fatigue, disorientation/confusion, swelling)	11.80%
**Total**		52.65%

Source: Medicare Fee for Service enrollment FY 2010/2011. Potentially preventable event rates computed for FY 2011.

APR DRG, All-Patient Refined Diagnosis-Related Group; EAPG, Enhanced Ambulatory Patient Groups; FY, fiscal year; NH, nursing home; PPA, potentially preventable hospital admissions; PPV, potentially preventable emergency department visits.

Three categories of EAPG–diseases and disorders of the digestive system, diseases and disorders of the kidney and urinary tract, and other factors influencing health status–contain 52.65% of PPVs. Various infections (eg, gastrointestinal, urinary tract infection) comprise approximately half of the PPVs in these categories.

**FIG. 1.**
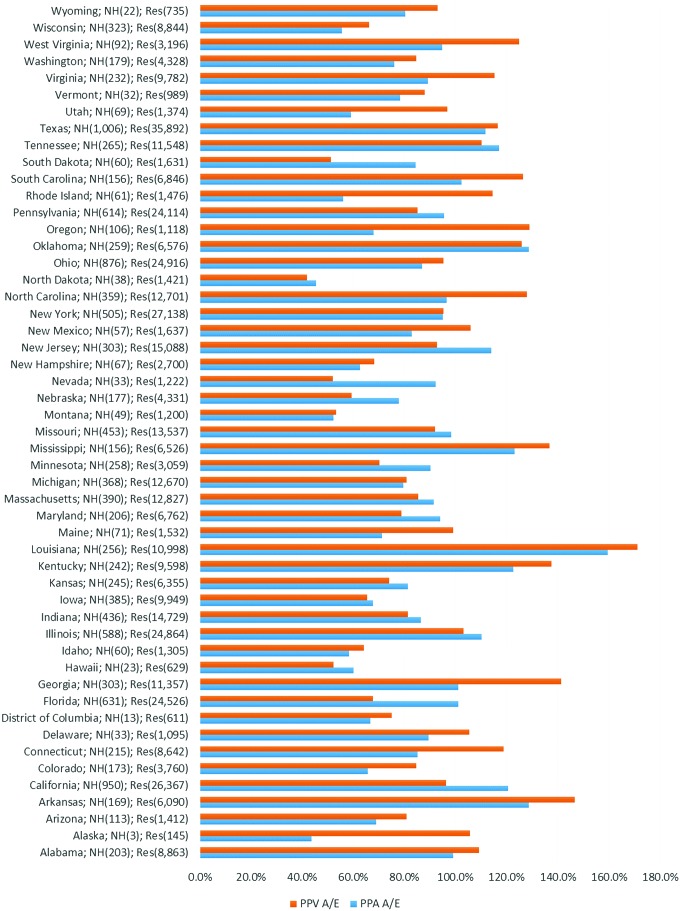
Distribution of nursing homes and residents with actual and expected PPA and PPV weights by state. Source: Medicare Fee for Service enrollment FY 2010/2011. Potentially preventable event rates computed for FY 2011. A/E, adjusted to expected ratio; FY, fiscal year; NH, number of nursing homes; PPA, potentially preventable hospital admission; PPV, potentially preventable emergency department visit; Res, number of nursing home residents. Color images are available online.

Figure 1 compares nursing home PPA and PPV rates by state for the 12,883 nursing homes and 439,011 beneficiaries. Aggregate PPA and PPV ratios are calculated by weighting state performance by the volume of nursing home residents. Figure 1 highlights the considerable performance variation across states. Nineteen states have a PPA ratio below 80% (better performance) of the national average and 15 states have a PPV ratio below 80%. Conversely 6 states have a PPA ratio above 120% (worse performance) and 10 have a PPV ratio above 120%. Of these, 9 states have (both) PPA and PPV ratios below 80% while 5 states have PPA and PPV ratios above 120% of the national average. Underlying this distribution the study team found that the worst performers (more PPA and PPVs per resident) were drawn from either the East or West South Central census regions with the worst performer, Louisiana, having the highest nursing home ratios for both PPA and PPV. Louisiana nursing homes have been identified previously as poor performers by the OIG,^15^ with nursing home hospitalization rates for the state 14 percentage points higher than the national average.

**FIG. 2.**
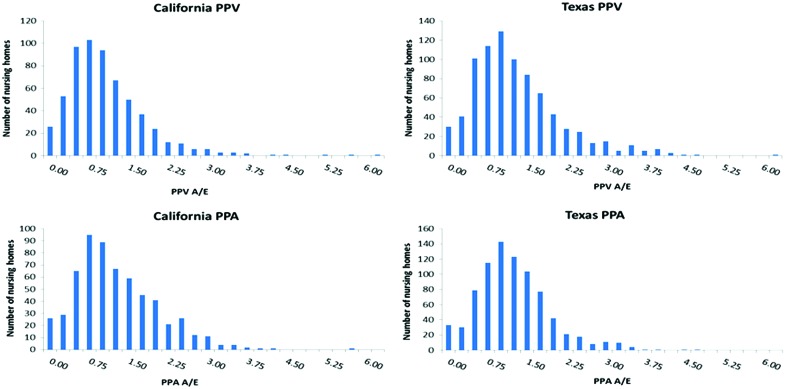
Distribution of PPA and PPV ratios for California and Texas nursing homes with more than 20 identified residents. Source: Medicare Fee for Service enrollment FY 2010/2011. PPA and PPV rates computed for FY 2011. N = 599 homes (22,325 residents) in California; 822 homes (33,632 residents) in Texas. A/E, adjusted to expected ratio; FY, fiscal year; PPA, potentially preventable hospital admissions; PPV, potentially preventable emergency department visits. Color images are available online.

Measurement of correlation between PPA and PPV ratios within states yields a significant Pearson correlation coefficient of r = .64, which exceeds the 99% confidence level. Figure 2 compares the range of nursing home performance in managing PPA and PPV events in 2 states: Texas and California. To reduce volatility only nursing homes with a minimum of 20 identified residents are included. Thus Figure 2 presents results for 599 nursing homes in California (22,325 beneficiaries) and 822 nursing homes in Texas (33,623 beneficiaries).

Variation across nursing home performance (Figure 2) is pronounced for both PPAs and PPVs. In California, 120 nursing homes (20%) perform at a PPA rate less than half that of the national expected rate while 83 (14%) perform at a rate twice that of the national PPA rate. The corresponding numbers for PPV are 176 (29%) and 48 (8%). Similar patterns are observed for Texas.

To compare results obtained from PPA and PPV analysis to the Nursing Home Compare Star rankings, the study team assigned each nursing home from all states the average Star ranking reported for the preceding 12 months. Nursing homes with fewer than 20 identified residents and those that had no Star ranking assigned were excluded. This resulted in the retention of 7972 nursing homes. Although the 3 components of Star rankings are integers, averaging the components resulted in overall Star values between integers for many homes. The team formed groups containing ranges of Star values beginning with 1 star established as the lowest level of quality; this range contained 955 nursing homes. Having elected to balance the distribution of nursing homes to ranges to obtain similar numbers of nursing homes within each range, the team created 8 Star ranges for the 7972 nursing homes. Nursing homes were assigned a rank based on their PPA and PPV ratios from 1 (highest ratio = worst performer) to 8 (lowest ratio = best performer). For each range the study team computed the associated mean rank and actual/expected ratio for PPAs and PPVs.

Table 3 shows that the average rankings under PPA and PPV broadly stay within the 4–5 range where mean ranking is 4.59. If differences in PPV and PPA ratios were reflected well within the Star rankings one would expect the mean rankings for the groups to rise from 1 to 8. Using the ordinal nursing home rankings for each measure, the study team calculated the degree to which the rankings concur using Kendall's Tau b statistic for concordant and discordant paired ranks. The resulting Kendall's Tau b statistics indicate significant but weak statistical association between the Star rankings and PPA (.086) and PPV (.124) measures.

**Table 3.** Comparison of Nursing Home Compare Five-Star Rating with Potentially Preventable Hospital Admission and Potentially Preventable Emergency Department Visit Ratios by Nursing Home

*Average CMS stars*	*Nursing homes*^a^	*Mean of PPA rank*^b^	*Mean of PPA ratio*^c^	*Mean of PPV rank*	*Mean of PPV ratio*
1	955	4.15	1.11	3.72	1.27
>1 < 2	991	4.26	1.07	4.12	1.14
≥2 < 2.3	1022	4.43	1.05	4.26	1.11
≥2.3 < 3	890	4.60	1.00	4.46	1.04
≥3 < 3.3	976	4.70	0.99	4.84	0.94
≥3.3 < 4	837	4.64	0.99	4.87	0.91
≥4 < 4.3	1268	4.93	0.92	5.09	0.85
>4.3	1033	4.91	0.94	5.22	0.83
**ALL** [2.88]	7972	4.59	1.01	4.59	1.01
**Kendall's Tau b CMS Stars**		.086^*^		.124^*^	

^*^ Significant association at the 99% 1-tailed level.

^a^ Excludes nursing homes for which a 5-Star ranking was not computed.

^b^ PPA or PPV rank = nursing home performance range from 1 (worst) to 8 (best).

^c^ PPA or PPV ratio = nursing home performance measure of actual/expected (lower ratio is better).

CMS, Centers for Medicare & Medicaid Services; PPA, potentially preventable hospital admissions; PPV, potentially preventable emergency department visits.

## Discussion

This analysis identifies considerable variation in the frequency of admissions (PPA) and ED visits (PPV) experienced by nursing home residents both across and within states, with high correlation between PPA and PPV rates within states (Figure 1). This variation persists after risk adjustment.

Figure 2 demonstrates the high level of PPA and PPV performance variability across nursing homes within states, such that poorly performing states have pockets of good performance and the reverse being true that better performing states have pockets of poor performers. Table 3 reports the weak statistical association between the Star rankings and the PPA and PPV measures. These findings raise several concerns about Nursing Home Compare Star rankings.

A first concern is that state-specific survey findings represent a significant portion of nursing home Star assignment. The assignment of 1 to 5 stars is first determined by relative ranking within a state on that state's surveys (ie, how nursing homes compare to other nursing homes within the state). A description of the Nursing Home Compare Star ranking method is given in the online Supplementary Materials (Supplementary Data). The initial ranking may only be modified by plus or minus 1 star for each of the staffing and quality metrics. The Government Accountability Office (GAO) recently recommended constructing a national comparison tool rather than having rankings driven by state-specific results,^16^ a position that CMS rejects.

Second, stars for staffing hours per resident (staffing metric) uses self-reported nursing home staffing data adjusted for case mix using the Resource Utilization Group resident classification system (used by CMS to pay skilled nursing facilities [SNF] under the SNF prospective payment system^17^), a process that has been highlighted by the GAO as a weakness for objective quality evaluation.^18^ The absence of data audits has contributed to a general disbelief in the accuracy of the reported staffing data coming from regulators and the nursing home industry itself.^19^ In addition, others have challenged the perceived link between a count of staffing hours and outcomes quality.^20,21^ Little distinction is made in the Compare calculation between long-term and short-term residents, further complicating the perceived relationship between staffing hours and long-term nursing home quality. The domination of Star rankings by staffing and, in particular, state surveys relative to quality metrics has led to accusations that Star rankings are “misleading and disingenuous,” with 20% of 4- or 5-star nursing homes having only 1 or 2 quality stars.^22^ These concerns are echoed in recommendations put forth by the OIG to place more emphasis on quality in general and, specifically, to consider the rate of hospital admission for nursing home residents.^15^

PPA and PPV results (Table 2) raise a third concern. Quality scores (stars) are calculated using a mix of measures applicable to both long-term residents (9 measures) and short-term residents (7 measures). Performance on each measure is made on a relative performance basis (typically using quintiles) with scores falling within a range used to assign stars. The serious and preventable conditions occurring in nursing homes, at highly variable rates, leading to admissions and ED encounters are, with the exception of urinary tract infections, absent from the list of measures in the Compare quality Star rankings; even for urinary tract infection, there is no adjustment for the mix of residents that a nursing home cares for. The emphasis placed on infection control for nursing home requirements of participation in the CMS modernization of nursing home oversight^23^ makes this an important gap to fill.

This is not to set aside the valuable insights offered by the long-term resident measures such as the use of restraints, pain management, use of antipsychotics, and pressure ulcers. In addition, however, nursing home quality metrics should incorporate the serious nature of preventable hospital and ED encounters; such information should be provided to nursing home residents (and their families), and be included within the Star ranking system. This can be achieved by either incorporating performance on these measures or by expanding the quality domain to focus on the infections that result in hospitalizations and ED visits.

Several limitations were encountered in conducting this analysis. First, the absence of a direct link between the nursing home and nursing home residents is a significant obstacle. The study team identified 439,011 residents in the analysis but would prefer to have a proper accounting of the full complement of 1,392,000 nursing home residents. The team believes that the matching approach taken has resulted in a reasonable representation of residents and sampling of nursing homes; however, this would be strengthened by routine coordination of Medicare claims data with dual benefit Medicaid enrollees. Second, the study focuses on dual benefit enrollees who are enrolled in Medicare FFS and omits those residents residing in nursing homes that are either ineligible for Medicare or enrolled with Medicare under alternative coverage to FFS. This necessarily limits the sample size when determining nursing home performance. Third, although the Nursing Home Compare rating system has added several quality parameters since the time of this analysis, the use of state-specific surveys continues to have the largest impact on the Star Rating weights.

The financial imperatives of subsidizing nursing beds, for which Medicaid has been the major source of reimbursement, have contributed to changes in the characteristics of nursing home residents and to the services they are offered. Nursing homes have been increasingly attempting to attract better paying Medicare patients with services oriented to rehabilitation and more emphasis on skilled services geared toward younger, less dependent patients^24^ who will be able to return home. In addition, there have been ongoing attempts, through programs such as the federally funded Money Follows the Person, to integrate more nursing home residents back into the community,^25^ but so far success has been highly variable across nursing homes.^26^ Despite these efforts, the average age of assisted living residents is now estimated at 87 years, and they are characterized by greater frailty and clinical complexity.^24^ So although the need to cater to younger shorter term residents and increasing reliance on filling skilled nursing beds has grown, the core need to manage nursing home quality with metrics such as those employed by this study remains.

Much attention has been placed on the performance of SNFs, often by referring hospitals, to help reduce 30-day hospital readmissions for which hospitals may be penalized. Studies have shown that, even given the need for nursing facilities to fill their skilled beds, there are barriers to implementing quality initiatives that otherwise would encourage hospitals to refer patients.^27^ Facing limits on responsiveness and financial penalties, hospitals are seeking other methods through which to select higher quality nursing facilities, such as through establishing preferred SNF networks.^28^ Although these quality initiatives focus on outcomes for a patient population that skews younger, is more likely to receive rehabilitation therapy, and is less medically frail and complex, the need to track and intervene in patient outcomes applies to all beds in the facility.

## Conclusion

Using a large national sample, this study found significant rates of PPAs and PPVs to be influenced by the chronic illness burden of nursing home residents, but with considerable variation in the frequency of these events across nursing homes, both within and across states.

Comparison of nursing home PPA and PPV performance to Star rankings yielded only a weak correlation. The study team believes that the Star rankings could benefit by incorporating performance on a subset of conditions that result in hospitalizations and ED visits for large numbers of residents, either by direct reporting of the events or by greater focus on those infections that cause them. The Star rankings should have greater emphasis on the outcomes of care within the quality domain so that the Nursing Home Compare Stars can better help users find nursing homes of higher quality and stimulate homes to improve quality in ways that benefit residents.

## Supplementary Material

Supplemental data
