# Supplementary material for: Nursing Home Compare Star Rankings and the Variation in Potentially Preventable Emergency Department Visits and Hospital Admissions
Source: Popul Health Manag. 2019 Mar 27;22(2):144–52. doi: 10.1089/pop.2018.0065 (PMC6459266; doi:10.1089/pop.2018.0065)
Supplement: Supplemental data [file Supp_Data.pdf]

Supplementary Data

Nursing home quality measures (QM) used in nursing home compare 5 star calculation

MDS* Based Long-Stay Measures
Percentage of residents whose ability to move independently worsened
Percentage of residents whose need for help with activities of daily living has increased
Percentage of high-risk residents with pressure ulcers
Percentage of residents who have/had a catheter inserted and left in their bladder
Percentage of residents who were physically restrained
Percentage of residents with a urinary tract infection
Percentage of residents who self-report moderate to severe pain
Percentage of residents experiencing one or more falls with major injury
Percentage of residents who received an antipsychotic medication
**MDS Based Short-Stay Measures**
Percentage of residents whose physical function improves from admission to discharge
Percentage of residents with pressure ulcers that are new or worsened
Percentage of residents who self-report moderate to severe pain
Percentage of residents who newly received an antipsychotic medication
**Claims-Based Short-Stay Measures**
Percentage of residents who were re-hospitalized after a nursing home admission
Percentage of short-stay residents who have had an outpatient emergency department (ED) visit
Percentage of short-stay residents who were successfully discharged to the community

*MDS, Minimum data set.

The overall star ranking, the integration of the three domains, is calculated by a simple three step process. First, set the star level indicated by the survey metric. Add a star (max of 5) if the staffing metric is rated at 4 or 5 stars, or deduct a star if the rating is 1 star. To this result add a star (max of 5) if the quality metric is rated as 5 star or deduct a star if the rating is 1. It can be seen from this that the star rating system is most heavily influenced by the survey metric (which is the stated intent).
